# The association between body dissatisfaction, depression, eating disorders and BMI: a prospective twin cohort study

**DOI:** 10.1192/j.eurpsy.2025.371

**Published:** 2025-08-26

**Authors:** I. Costantini, T. C. Eley, N. M. Davies, H. Bould, C. M. Bulik, G. Krebs, P. Diedrichs, G. Lewis, G. Lewis, C. Llewellyn, D. Nicholls, J.-B. Pingault, F. Solmi

**Affiliations:** 1 Division of Psychiatry, University College London; 2 Social, Genetic and Developmental Psychiatry Centre, Institute of Psychiatry, Psychology & Neuroscience, King’s College, London; 3 Population Health Sciences, University of Bristol, Bristol, United Kingdom; 4 Department of Psychiatry, University of North Carolina, Chapel Hill, United States; 5 Research Department of Clinical, Educational and Health Psychology, University College London, London; 6 Centre for Appearance Research, University of the West of England, Bristol; 7 Department of Behavioural Science & Health, University College London; 8 Division of Psychiatry, Imperial College London; 9 Department of Clinical, Educational and Health Psychology, Division of Psychology and Language Sciences, University College London, London, United Kingdom

## Abstract

**Introduction:**

Body dissatisfaction is becoming more common among adolescents and is a putative risk factor for adverse mental (e.g., eating disorder and depressive symptoms) and physical health outcomes (e.g., excessive weight gain), both of which have also been increasing. Targeting body dissatisfaction through preventative interventions might improve these outcomes, however robust evidence of causal associations is limited.

**Objectives:**

To investigate the association between body dissatisfaction at age 16 and eating disorder symptoms at age 21, as well as depressive symptoms and BMI at ages 21 and 26 using a co-twin control design.

**Methods:**

We used data from the Twins Early Development Study (TEDS) and validated self-report measures. We fitted univariable and multivariable linear mixed effect models adjusting for a comprehensive list of confounding factors (Figure 1) to investigate the association between body dissatisfaction, eating disorder and depressive symptoms, and BMI in the full twin sample. We then repeated these analyses using a co-twin control design, which allows to fully and partially control for genetic confounding in monozygotic (MZ) and dizygotic (DZ) twins, respectively, and for any shared measured and unmeasured environmental factors. We conducted primary analyses in imputed datasets for participants with complete exposure data.

**Results:**

The analytical sample included 2,183 twins (60.2% females, 61.7% DZ twins). In the full twin sample, one unit increase in body dissatisfaction at age 16 was associated with: (i) a 1.80-point increase in eating disorder symptoms at age 21 (95%CI: 1.49 to 2.11), (ii) a 0.59-point increase in depressive symptoms (95%CI: 0.46 to 0.72), and (iii) a 0.20-point BMI increase (95%CI: 0.08 to 0.32) across age 21 and 26 years. In co-twin control analyses, the association between body dissatisfaction and eating disorder symptoms was larger in DZ twins (N=661; 1.72, 95%CI: 1.11 to 2.33) than in MZ twins (N=414, 0.96, 95%CI: 0.15 to 1.77), whereas effect sizes for depressive symptoms were comparable [DZ: (0.56, 95%CI: 0.33 to 0.78); MZ: (0.50, 95%CI: 0.15 to 0.85)]. Associations with BMI were smaller in DZ (0.20, 95%CI: 0.00 to 0.40), and null in MZ twins (0.07, 95%CI: -0.21 to 0.35).

**Image 1:**

**Figure:**
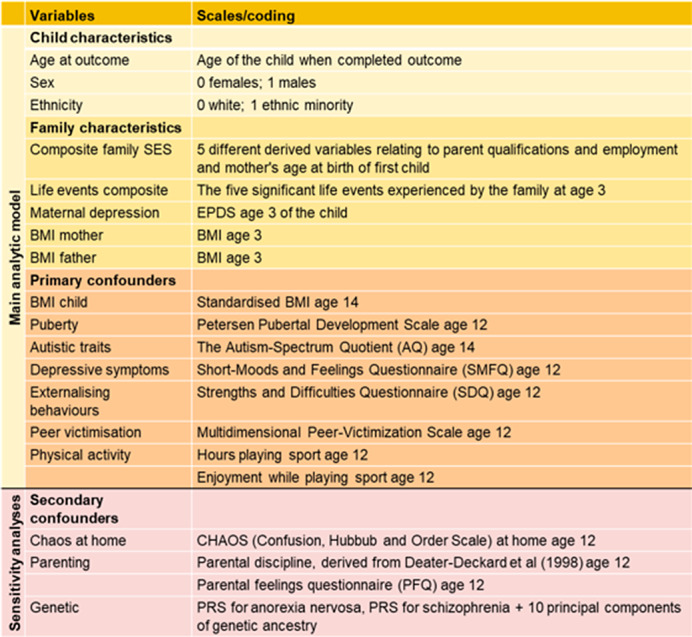

**Conclusions:**

Our findings suggest that greater body dissatisfaction might be a causal risk factor for eating disorders and depression in young people, as associations seen in the full sample persisted in co-twin control analyses. This indicates that body dissatisfaction could be a modifiable target to reduce the risk of these mental health problems in adolescents and young adults. Evidence of associations between body dissatisfaction and increased BMI was weaker in co-twin control analyses than in the full sample. This might be due to larger proportions of shared genetic risk and thus require larger sample sizes to detect.

**Disclosure of Interest:**

None Declared

